# Acupuncture for radiation-induced toxicity in head and neck squamous cell carcinoma: a systematic review based on PICO criteria

**DOI:** 10.1007/s00405-021-07002-1

**Published:** 2021-07-31

**Authors:** Pierluigi Bonomo, Giulia Stocchi, Saverio Caini, Isacco Desideri, Veronica Santarlasci, Carlotta Becherini, Vittorio Limatola, Luca Giovanni Locatello, Giuditta Mannelli, Giuseppe Spinelli, Carmelo Guido, Lorenzo Livi

**Affiliations:** 1 Radiation Oncology, Azienda Ospedaliero-Universitaria Careggi, University of Florence, largo Brambilla 3, 50134 Florence, Italy; 2 Cancer Risk Factors and Lifestyle Epidemiology Unit, Institute for Cancer Research, Prevention, and Clinical Network (ISPRO), Florence, Italy; 3 Integrative Medicine Unit, Department of Anesthesia and Intensive Care, Azienda Ospedaliero-Universitaria Careggi, Florence, Italy; 4 Department of Otorhinolaryngology-Head and Neck Surgery, Azienda Ospedaliero-Universitaria Careggi, Florence, Italy; 5 Head and Neck Oncology and Robotic Surgery, Department of Experimental and Clinical Medicine, Azienda Ospedaliero-Universitaria Careggi, Florence, Italy; 6 Maxillo Facial Surgery Unit, Azienda Ospedaliero-Universitaria Careggi, Florence, Italy; 7 Fior Di Prugna Center for Complementary Medicine, Azienda USL Toscana Centro, Florence, Italy

**Keywords:** Head and neck cancer, Radiotherapy, Chemotherapy, Acupuncture, Toxicity

## Abstract

**Purpose:**

In head and neck squamous cell carcinoma (HNSCC), the potential mitigating effect of complementary medicine interventions such as acupuncture for radiation-induced toxicity is unknown. This study aimed to assess the impact of acupuncture on the incidence and degree of severity of common radiation-induced side effects.

**Methods:**

In accordance with pre-specified PICO criteria, a systematic review was performed. Two electronic databases (Medline and Embase) were searched over a 10-year time frame (01/01/10 to 30/09/20). Patients undergoing a curatively intended, radiation-based treatment for histologically confirmed squamous cell carcinoma of the nasopharynx, oropharynx, larynx, hypopharynx and oral cavity represented the target population of our study. Accurate information on the acupuncture methodology was reported. All included articles were evaluated to identify any potential source of bias

**Results:**

Five papers were included in our qualitative analysis, for a total of 633 subjects. Compliance to per-protocol defined schedule of acupuncture sessions was high, ranging from 82 to 95.9%. Most patients (70.6%) were randomly allocated to receive acupuncture for its potential preventive effect on xerostomia. The large heterogeneity in study settings and clinical outcomes prevented from performing a cumulative quantitative analysis, thus no definitive recommendations can be provided.

**Conclusions:**

Although shown to be feasible and safe, no firm evidence currently supports the use of acupuncture for the routine management of radiation-induced toxicity in HNSCC.

## Introduction

Head and neck squamous cell carcinoma (HNSCC) encompasses a heterogeneous group of primary tumors with distinct histologic features and clinical behavior that arise from the oral cavity, oropharynx, hypopharynx, larynx and nasopharynx, accounting for 6% of all malignant neoplasms [1]. At diagnosis, about 60% of patients have a loco-regionally advanced disease [2], potentially amenable to curatively intended management. Often in combination with cisplatin-based concurrent chemotherapy (CT), radiotherapy (RT) represents a mainstay of treatment for the majority of these late-stage presentations, either as definitive modality or after surgery [3]. Alongside the recognition of the prominent role of RT in HNSCC management, the delivery of radiation has historically been challenged by the known complexity of head and neck anatomy, whereby it is paramount that radiation oncologists aim for the right balance between pursuing a tumoricidal effect and avoiding undue toxicity. The inadvertent dose accumulation to unaffected oral and pharyngeal mucosal surfaces, salivary glands, teeth, mandible, larynx, skin, and soft tissue of the neck, may be associated with the development of side effects, both short and long terms. Conventionally regarded per Common Terminology Criteria for Adverse Events (CTCAE) definition [4] as occurring within 3 months from the end of treatment, radiation-induced acute toxicity entails the onset of oral mucositis [5], dysphagia [6], dysgeusia [7], salivary impairment [8], and dermatitis [9], posing a threat to the safe delivery of prescribed RT dose intensity and patient’s compliance. Late complications, such as xerostomia [10], trismus [11], osteoradionecrosis of the jaw [12], generalized fibrosis and lymphedema [13], muskuloskeletal disorders [14] and dental pathologies [15], may be prohibitive as well, with marked detriment to quality of life [16, 17] and social functioning [18] of HNSCC survivors. In addition, non-cancer-related mortality [19] can be considered as a potential treatment-induced late event, particularly in regards to aspiration pneumonia occurring after pharyngo-laryngeal dysfunction due to concurrent chemo-radiotherapy (CRT) [20, 21]. Thanks to its exquisite dose distribution allowing for improved sparing of organs at risk over conventional three-dimensional conformal techniques, Intensity-modulated radiation therapy (IMRT) was firmly established as standard practice for all patients with HNSCC [22]. Together with evidence-based demonstration of IMRT benefit [23], fostering multidimensional supportive care measures [24, 25] and implementing survivorship programs [26] have been more recently outlined as key care strategies. However, optimizing the management of patients developing radiation-induced acute and late toxicities is still an unmet need in head and neck cancer. Within the scope of complementary medicine the use of acupuncture for symptom control in oncology [27–29] has been increasingly advocated over the last two decades. Being recognized as an integral component of traditional Chinese medicine, acupuncture represents a complex nonpharmacologic intervention with a very favorable tolerability profile: briefly, it is characterized by the repeated application of thin metallic needles in discrete points of human anatomy (“acupoints”) and their ensuing manipulation with the purpose to modulate brain regions involved in cognition and emotion. Currently, its potential mitigating effect is part of the therapeutic armamentarium for common systemic therapy-related side effects, such as nausea and vomiting [30], fatigue [31], chronic pain [32], peripheral neuropathy [33, 34], and arthralgia [35]. In the context of head and neck cancer, the results of a randomized phase 2 study published in 2010 [36] and subsequent observations [37, 38] lend support to the application of acupuncture mainly for symptoms occurring after neck dissection, such as shoulder pain and arm dysfunction. Concerning common radiation-induced side effects, the available evidence is sparse, hampering definitive recommendations in the clinic. Since the non-surgical management of locally advanced HNSCC is usually associated with a narrow therapeutic index and debilitating consequences, we aimed to address whether the use of acupuncture can be recommended for patients primarily treated with RT. In addition, we sought to analyze its potential impact on the occurrence and degree of severity of distinct radiation-induced side effects.

## Materials and methods

In accordance with the Preferred Reporting Items for Systematic Reviews and Meta-Analyses (PRISMA) statement [39], a systematic review of the literature was conducted. Two electronic databases (Medline and Embase) were searched over a 10-year time frame (01/01/10 to 30/09/20). The MesH search strategy was as follows: PubMed search strategy, “head and neck radiotherapy AND acupuncture”(“Head and Neck Neoplasms”[Mesh]) AND (“Radiotherapy”[Mesh]) AND “Acupuncture Therapy”[Mesh]; EMBASE search strategy, (‘head and neck tumor’/exp OR ‘head and neck tumor’) AND (‘radiotherapy’/exp OR radiotherapy) AND (‘acupuncture’/exp OR acupuncture) AND [2010–2020]/py AND [english]/lim. Case reports, case series with less than 20 patients, reviews and consensus statements were not included. Only full-text papers available in English could be evaluated. The reference lists of the reviewed articles were manually searched. Conference proceedings of main international conferences (ASCO, ASTRO, ESMO, and ESTRO) were also analyzed; however, data published in abstract form only were excluded. As a guidance for the search strategy, the following PICO criteria [40] were addressed:

### Population

Patients undergoing a curatively intended, radiation-based treatment for histologically confirmed squamous cell carcinoma of the nasopharynx, oropharynx, larynx, hypopharynx and oral cavity represented the target population of our study. The use of CRT or RT as adjuvant therapy after primary surgery or as palliative treatment for recurrent/metastatic disease was not allowed. Subjects with HNSCC of any other primary anatomic location in the head and neck or non-squamous histologies were not eligible.

### Intervention

Reporting accurate information on the acupuncture methodology was mandatory upon inclusion of reviewed articles in our analysis. Details on the total number and individual duration of acupuncture sessions, the needle map insertion protocol, the use of sham technique as control arm and the degree of experience of board-certified acupuncturists were retrieved, whenever available. The timing of acupuncture application in respect to RT was also described, whether performed before, during or after its completion. Alternative techniques such as acupuncture-like transcutaneous electrical nerve stimulation (ALTENS) and combination acupuncture not complying with the Chinese traditional manipulation were not allowed.

### Comparators

In view of their potential impact on the development of side effects, treatment-related features were defined as “comparators”. In respect to RT, data on technique (3DCRT or IMRT), total dose, fractionation, dose correlates to relevant organs at risk such as parotid glands and pharyngeal constrictor muscles and treatment delays (elapsed days) were collected, whenever available. When systemic therapy was prescribed in combination with radiation, either one of the following options was categorized:

- 3-weekly cisplatin (100 mg/m^2^)
- weekly cisplatin (40 mg/m^2^)
- cetuximab (loading dose of 400 mg/m^2^, weekly dose of 250 mg/m^2^)
- other

In all cases, information on the mean relative dose intensity (RDI, defined as the delivered percentage of the total planned dose) and median number of administered cycles were extrapolated, whenever available. In case induction chemotherapy was prescribed before RT, the same data were retrieved. In addition, qualitative descriptors of supportive measures (such as the use of prophylactic percutaneous endoscopic gastrostomy, early implementation of palliative care, hospitalization rate, etc.) were searched in the reviewed articles.

### Outcomes

The potential impact of acupuncture on treatment-related side effects was differentiated based on their time of onset. By definition, acute toxicity referred to complications occurring during treatment or within 90 days from the end of radiation, whereas late toxicity to those developing thereafter. As per CTCAE [4], the following were the most common adverse events to be searched for: nausea, vomiting, dysgeusia, oral mucositis, radiation dermatitis, dysphagia, pain, fibrosis and xerostomia. The rates of any-grade and severe (G3–G4) toxicity were reported, if specified. The correlation between the use of acupuncture and health-related quality of life through standardized patient-reported outcomes was also explored, whenever available. Articles focused on surgical complications such as shoulder pain or dysfunction following neck dissection were not considered.

### Statistical analysis

Baseline patient characteristics, disease and treatment features were summarized using descriptive statistics (mean, median and frequency distribution). In accordance with the Cochrane Review tool [41], all included articles were evaluated to identify any potential source of bias. In particular, the presence of bias was assessed in terms of risks of selection (random sequence generation), reporting (selective outcome reporting), performance (knowledge of the allocated interventions), detection (lack of precise definition and reliable method to detect and report the outcome), and attrition (deviations or handling of incomplete outcome data), respectively. Initially, we had planned to combine study-specific estimates of relative risks (risk ratio-RR or odds ratio-OR) for the effect of acupuncture on the incidence of treatment-related side effects into summary relative risks using random effects meta-analysis models, and to quantify the between-studies heterogeneity using the *I*^2^ statistics. However, the small number and low comparability of eligible studies prevented us from performing this kind of approach.

## Results

### Data collection and analysis

**Fig. 1:**
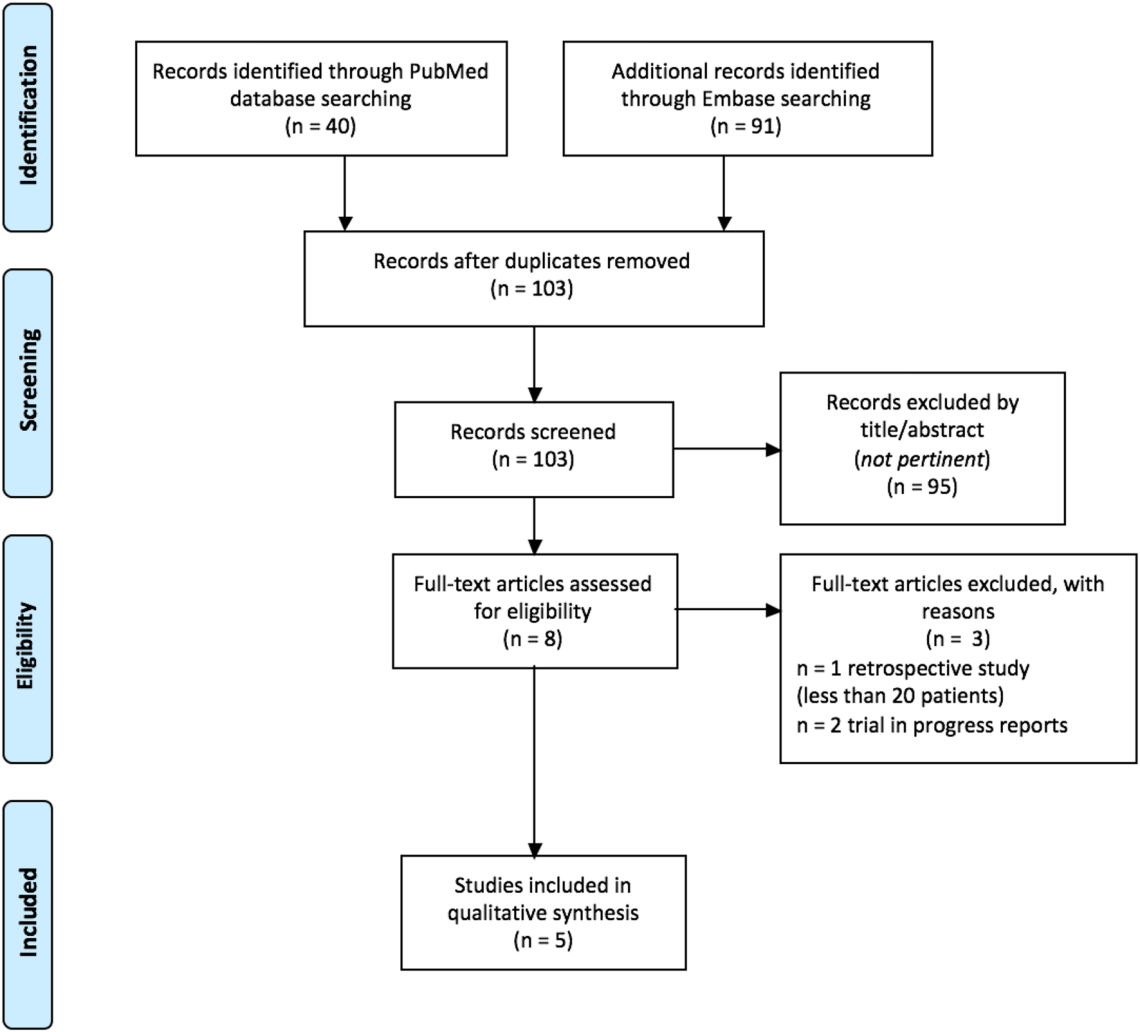
Flow chart of literature search

**Fig. 2:**
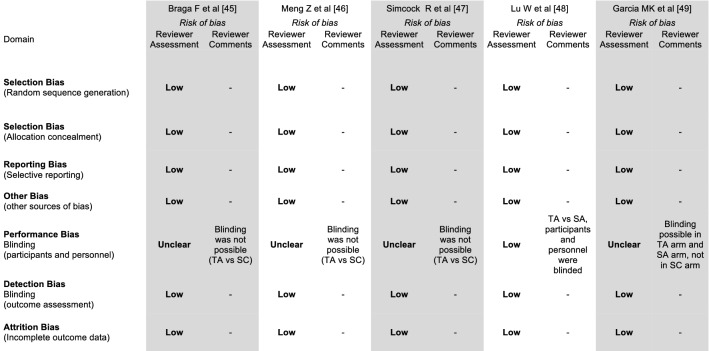
Quality assessment according to the Cochrane review tool. *TA* true acupuncture; *SA* sham acupuncture; *SC* standard care

An independent assessment of the reviewed literature was performed by two authors (PB and GS). The identified references were analyzed through a data collection sheet. Discrepancies were resolved by consensus with a third author (ID). By applying our prespecified search criteria, a total of 151 articles was retrieved (Fig. 1). After removing duplicates, 103 papers were screened through abstract assessment, 8 of whom qualified for full-text analysis. Homb et al. [42] performed a retrospective analysis of combination acupuncture for xerostomia on 16 patients, thus below the prespecified threshold of 20 subjects upon inclusion in our study. For the same reason, the interim-report [43] on first 15 patients enrolled in a randomized trial assessing the impact of acupuncture on acute toxicity during CRT was also excluded, whereas the publication on the controlled, randomized Rosetta trial [44] had no patient information. Overall, 5 articles published between 2011 and 2019 fulfilled our inclusion criteria and were retained in the final analysis (Table 1). All were performed prospectively and had a randomized design, except for Braga’s case–control cohort study [45]. In terms of Cochrane review tool assessment (Fig. 2), methods of random sequence generation and allocation of patients to treatment groups were specified in all included articles; therefore, a low risk of selection bias was assigned. No selective reporting of outcomes could also be detected. In addition, the attrition bias was deemed low as outcome data were found to be complete. In two studies [48, 49], a sham acupuncture (SA) arm was designed, allowing for blinding between treatment groups. Overall, despite the heterogeneity of the study population in the included articles and the variety of assessment scales for the main outcomes, an overall low risk of bias could be found.

**Table 1:** Study design and patient characteristics

Author [ref]	Year	Study design	Patients (total)	Setting	Patients (treated with TA)	Patients (treated with SA)	Patients (treated with SC)	Age, years (mean)	Age, years (range)	Male no. (%)	Female no. (%)
Braga F et al. [45]	2011	Prospective cohort study	24	single center	12	0	12	63	44–82	16 (66.7)	8 (33.3)
Meng Z et al. [46]	2012	Randomized phase 2 study	84	single center	39	0	45	47.2	ns	59 (70.2)	25 (29.8)
Simcock R et al. [47]	2013	Randomized crossover phase 3 study	144	multicenter (7)	144^a^	0	144^a^	59.4	41–83	109 (75.7)	35 (24.3)
Lu W et al. [48]	2016	Randomized phase 2 study	42	multicenter (2)	21	21	0	58.1	ns	34 (80.9)	8 (19.1)
Garcia MK et al. [49]	2019	Randomized phase 3 study	339°	multicenter (2)	112	115	112	51.3	21–79	258 (77.6)	81 (22.4)

339 patients were included in the final analysis out of 399 randomized subjects

*TA* true acupuncture, *SA* sham acupuncture, *SC* standard care

^a^74 patients were randomized to oral care followed by TA, whereas 70 to TA followed by oral care

### Patient, disease and treatment-related features

A total of 633 patients was included in our analysis, of whom 328 (51.8%), 313 (49.4%) and 136 (21.4%) received true acupuncture (TA), standard care (SC) and SA, respectively (Table 1). Of note, Simcock et al. [47] designed a randomized, cross-over phase 3 study, whereby all 144 enrolled subjects received TA, either anticipated (74/144, 51.3%) or followed (70/144 48.3%), by standard oral care. Most patients were male (476/633, 75.1%), with a mean age of 55.8 years (overall range across studies, 21–83). Despite the fact that in all studies, it was acknowledged that patients were in good clinical conditions and deemed candidate to receive a curatively intended treatment, no formal description of performance status could be retrieved from the included papers. In addition, no data were reported in terms of smoking exposure and pre-existing comorbidities. The two most common primary tumor sites were nasopharynx and oropharynx, with 314 (49.6%) and 270 (42.6%) cases each, respectively. No data could be found in regards to Human Papilloma Virus (HPV) status and Epstein Barr virus (EBV) detection. Although stage information was missing in two papers, most patients were treated for a locally advanced disease (combined stage III and IV, 403/484, 83.2%) (Table 2). In regards to treatment characteristics, more than a third of the whole patient population was irradiated with outdated techniques (two dimensional and 3DCRT: 251/652, 38.4%), whereas IMRT was used in the two most recent works [48, 49] (Table 3). RT prescription did not differ significantly among the included studies or between arms, with a mean total dose ranging between 63.5 and 70.9 Gy delivered through a standard 6–7 week schedule. In respect to RT dose distribution to critical organs at risk and potential correlation with toxicity, some insight was provided in 3 papers. In Braga’s [45] and Simcock’s [47] works, it was reported that a significant portion of parotid glands was included in the designed RT fields. In spite of IMRT use, the optimal constraint (mean dose below 26 Gy) for both parotids was not kept in over 60% of cases in Garcia’s trial [49]. Notably, the mean dose to parotid glands was a stratification factor in this large, three-arm randomized study. Dosimetric data on oral cavity and pharyngeal constrictor muscles were not available. In view of the prevalent advanced disease stage of the whole sample, as expected most patients (444/652, 68%) received concurrent CT, although no information could be retrieved in terms of type of systemic agent, number of cycles and RDI. No data could be found on RT interruptions either. With the exception of the ARIX trial [47], where recruited patients had a minimum diseas-free interval of 18 months from RT completion, in the remaining 4 works acupuncture was mainly administered throughout a standard 6–7 week RT schedule, with variable frequency. In all studies, board-certified, experienced acupuncturists applied the needles, following well-defined protocols for about 20 min per session. Overall, the completion rate of acupuncture sessions was very high, ranging from 82% [46] to 95.9% [49]. No or very rare adverse events (i.e., mild discomfort, minor site bruise) related to acupuncture were reported, with a crude incidence far below 5% of the whole population. In two studies [48, 49] SA was performed in accordance with standardized methodology. In both trials, quality audits of both TA and SA were carried out per protocol. With the exception of the ARIX trial [47], where an educational approach of two group oral care sessions was envisaged before or after TA in both arms, in the remaining papers no specific intervention in the SC groups was attempted to minimize RT-related side effects, outside of routine management. Critically, no information on supportive care measures adopted during or after RT could be retrieved from the included studies. Overall, two additional limitations can be condensed from the analyzed studies: first, 74/652 patients (11.3%) received some form of surgical manipulation before RT; however, as already mentioned, RT dose delivery was always in the curative range (> 60 Gy) for all cases. Second, the follow-up time was not uniformly completed, with 19%, 28% and 52.8% of missing data at predefined study timepoints in Lu’s [48], Meng’s [46] and Simcock’s [47] works, respectively.

**Table 2:** Disease features

Author [ref]	Year	Patients (total)	Primary tumor site no. (%)						Disease stage no (%)			
			Oral cavity	Oropharynx	Larynx	Hypopharynx	Nasopharynx	Other/unknown	I	II	III	IV
Braga F et al. [45]	2011	24	5 (20.8)	3 (12.5)	7 (29.2)	4 (16.7)	1 (4.1)	4 (16.7)	ns	ns	ns	ns
Meng Z et al. [46]	2012	84	0	0	0	0	84 (100)	0	5 (5.9)	27 (32.2)	34 (40.5)	18 (21.4)
Simcock R et al. [47]	2013	144	11 (7.7)	100 (69.4)	9 (6.2)	6 (4.2)	6 (4.2)	12 (8.3)	ns^a^	ns	ns	ns
Lu W et al. [48]	2016	42	1 (2.4)	30 (71.4)	2 (4.8)	1 (2.4)	2 (4.8)	6 (14.2)	1 (2.4)	0	5 (11.9)	36 (85.7)
Garcia MK et al. [49]	2019	358^a^	0	137 (38.3)	0	0	221 (61.7)	0	11 (3)	37 (10.4)	118 (33)	192 (53.6)

*ns* not stated

^a^Includes all participants with baseline data plus at least 1 follow-up at any time

^b^Only separate T and N data, no information on stage

**Table 3:** Treatment and acupuncture features

Author [ref]	Patients	RT technique	RT total dose (mean, Gy)	Dose to OAR’s (%)	Induction CT (n, %)	Concurrent CT (n, %)	Timing of acupuncture	Acupuncture sessions (n)	Acupoints protocol
Braga F et al. [45]	TA: 12 SC: 12	2D (all)	67.7 68.1	RT fields involved > 50% of major salivary glands (all)	/	4/12 (33.3) 7/12 (58.3)	before and during RT, twice a week	16–20	ST-3, ST-4, ST-5, ST-6, ST-7, GB-2, SI-19, TB-21, LI-4, LI-11, LR-3, ST-26, KI-3, KI-5, GV 20, Shen-Men, Centra Nervous System, Kidney, Spleen, Pancreas, Mouth 14 bilaterally + GV-20 (29 acupoints for session)
Meng Z et al. [46]	TA: 39 SC: 45	3DCRT (all)	70.8 70.9	no data provided	41/84 (48.8)	39/84 (46.4)	During RT, 3 times a week	21	Ren-24, Lung-7, Kidney-6, Shen-Men, Point Zero, Salivary Gland 2’, Larynx 6 bilaterally + Ren-24 (13 acupoints for session)
Simcock R et al. [47]	Oral care-TA: 74 TA-oral care: 70	2D/3D: 143 IMRT: 1 (all)	63.5 65.6	At least 1 parotid gland in the irradiated field (all)	/^a^	46/74 (62.1) 48/70 (68.5)	After RT, disease free-interval ≥ 18 months; once a week	8	Salivary Gland 2’, Modified Point Zero, Shen-Men, LI-2, LI-20 6 bilaterally (12 acupoints for session)
Lu W et al. [48]	TA: 21 SA: 21	IMRT (all)	69.2 68.7	no data provided	/^a^	42/42 (100)	During and after RT, starting 2 weeks into RT up to 20 weeks after it; once every 2 weeks	12	ST7, ST6, ST5, GB20, SI16, CV23, LI11, LI2, SP9, K3, ST36, SP6, GV20, GV24, Yintang, CV24 12 bilaterally (28 acupoints for session)
Garcia MK et al. [49]	TA: 118^b^ SA: 124 SC: 116	IMRT (all)	66–70.4 (prescribed dose; all)	78: PGs > 26 Gy^c^ (66) 79: PGs > 26 Gy^c^ (63) 79: PGs > 26 Gy^c^ (68)	69/118 (58) 77/124 (62) 72/116 (62)	86/118 (72.8) 91/124 (73.3) 81/116 (69.8)	During RT, 3 times a week	18–21	Salivary Gland 2’, Shen-Men, Point Zero, Larynx, LU7, K6, GB32, Ren-24 6 bilaterally (14 acupoints for session)

*RT* radiotherapy, *Gy* Gray, *OAR’s* organs at risk, *CT* chemotherapy, *TA* true acupuncture, *SA* sham acupuncture, *SC* standard care, *2D* 2-dimensional radiotherapy, *3DCRT* 3-D conformal radiotherapy, *IMRT* intensity modulated radiotherapy, *PGs* parotid glands

^a^Unspecified as whether chemotherapy was given before, concomitantly to radiation, or both

^b^Includes all participants with baseline data plus at least 1 follow-up at any time (358)

^c^Mean dose

### Impact of acupuncture on radiation-induced toxicity

The impact of acupuncture in mitigating radiation-induced side effects is summarized in Table 4. The potential preventive effect of TA on acute xerostomia (by definition, occurring up to 3 months from RT completion) was addressed in 2 papers. In a small, non-randomized case–control study, Braga et al. [45] showed a significant difference in terms of objective sialometry measures on the last RT session. This finding was somewhat validated by Meng et al. [46], who observed a persistent effect on stimulated salivary flow rate up to 6 months after treatment (RR 0.62, *p* < 0.003). In addition, symptoms related to severe parotid dysfunction were significantly less likely (RR 0.63, *p* = 0.019 and RR 0.38, *p* = 0.0018 at 1 and 6 months, respectively) when patients had been exposed to TA during radiation. More recently, a large, dual-institution 3-arm randomized phase 3 trial [49] demonstrated that a preventive effect can be exerted on 1-year salivary dysfunction, as well. In comparison to SC, TA was associated with significantly fewer and less severe patient-reported xerostomia symptoms. However, subtle, marginally significant differences emerged when comparing the effect of TA with SA, in line with known concerns available from the literature [50] on the reliability of using placebo needles as control arm. In addition to the preventive role of TA on the occurrence of xerostomia, the multicenter ARIX trial [47] addressed its potential symptomatic efficacy in patients with chronic salivary impairment after 3DCRT. Although a significant degree of relief was demonstrated 9 weeks after TA in respect of specific patient-reported domains, such as “dry mouth” (RR 2.01, *p* = 0.031) and “sticky saliva” RR (1.67, *p* = 0.048), no difference from an oral care educational session was observed in terms of overall quality of life and sialometry measures. Of note, the extremely variable timing between RT completion and randomization (median interval of 41 months, range 18–104 months) may have diluted the strenght of the intervention. In addition to xerostomia, a single small randomized phase 2 study [48] explored the potential preventive effect of TA on dysphagia 1 year after treatment. Other than demonstrating the feasibility of both TA and SA interventions, no significant difference emerged between the two arms in terms of dysphagia-related quality of life.

**Table 4:** Impact of acupuncture on radiation-induced toxicity, qualitative analysis

Author [ref]	Patients	Primary endpoint	Assessment	Main secondary endpoint	Assessment	Efficacy size	Main message
Braga F et al. [45]	TA: 12 SC: 12	RSFR SSFR	Last day of RT (mixed model procedure with random and fixed factors; ANOVA)	Patient-reported xerostomia through a modified xerostomia questionnaire (4-item VAS)	Last day of RT (mixed model procedure with random and fixed factors; ANOVA)	Primary: mean RSFR, 0.21 vs 0.04 mL/min; mean SSFR, 0.49 vs 0.12 mL/min (*p* < 0.001) Secondary: mean scores of 4-item VAS lower (Q1, Q2, Q4) and higher (Q3) with TA (*p* < 0.001)	Improved objective sialometry measures and decreased xerostomia-related symptoms at RT completion: preventive effect of TA on acute toxicity
Meng Z et al. [46]	TA: 39 SC: 45	Patient-reported xerostomia through Xerostomia Questionnaire (8-item)	1 and 6 months after RT (SAS PROC MIXED, linear mixed models; chi square-analyses)	RSFR SSFR	1 and 6-months after RT (SAS PROC MIXED, linear mixed models; analysis of covariance)	Primary: @1 month, 54.3%vs 86.1% of patients had XQ > 30 (RR 0.63; 95% CI 0.45–9.87, *p* = 0.019) @6 months, 24.1% vs 63.6% of patients had XQ > 30 (RR 0.38; 95% CI 0.19–0.76, *p* = .0018) Secondary: SSFR @6 months of 1.57 vs 0.95, group difference 0.62; 95% CI 0.22–1.01, *p* < 0.003)	Improved patient-reported severe xerostomia and time-trend effect on SSFR: preventive effect of TA on acute and early late (6-month) toxicity
Simcock R et al. [47]	Oral care-TA: 74 TA-oral care: 70	Patient-reported xerostomia through EORTC H&N 35 + 4 study-specific items	3 and 6 months after TA (logistic regression models)	Global QoL score RSFR SSFR	3 and 6 months after TA (logistic regression models)	Primary: @ week 9 after TA, improved “dry mouth” (H&N 35 Q41) (OR 2.01; 95% CI 1.38–2.64, *p* = 0.031) and “sticky saliva” (H&N Q.42) (OR 1.67, 95% CI 1.16–2.17, *p* = 0.048) Secondary: no difference	With a 4-week crossover randomized design between two interventions, TA improved symptom relief: symptomatic effect on chronic xerostomia
Lu W et al. [48]	TA: 21 SA: 21	Patient-reported dysphagia through MDADI (total and subscale scores)	Change from baseline to 12 months after RT/6 months after TA (repeated measures mixed model)	Feasibility	Final data analysis (descriptive measures)	Primary: @ 12 months, MDADI scores improved in both groups (+ 7.9 points in TA, 95% CI 0.2–15.6; + 13.9 points in SA, 95% CI 6.4–21.4) without any difference between TA and SA (*p* = .17, 95% CI − 14.7–2.7, SD 4.3)	Feasible trial with audit-confirmed high fidelity (96%) of TA and SA protocols; no difference between TA and SA in improving dysphagia-related QoL
Garcia MK et al. [49]	TA: 112 SA: 115 SC: 112	Patient-reported xerostomia through Xerostomia Questionnaire (8-item)	12 months after RT (analysis of covariance; mixed-model analyses of repeated measures)	Incidence of severe xerostomia (XQ > 30)	12 months after RT (analysis of covariance; mixed-model analyses of repeated measures)	Primary: @ 12 months after RT, improved mean XQ scores when comparing TA vs SC [adjusted least square mean XQ score of 26.6 (SD 17.7) vs 34.8 (SD 18.7), effect size -0.44, *p* = 0.001], no difference between TA and SA Secondary: @ 12 months after RT, less clinically severe xerostomia when comparing TA vs SC (unadjusted mean XQ score of 30 or more in 34.6% vs 55.1% of patients; *p* = 0.009)	Improved patient-reported xerostomia and less severe symptoms: preventive effect of TA on late xerostomia

*TA* true acupuncture, *SA* sham acupuncture, *SC* standard care, *RSFR* resting salivary flow rate, *SSFR* stimulated salivary flow rate, *ANOVA* analysis of variance, *VAS* visual analog scale, *RT* radiotherapy, @ at, XQ xerostomia questionnaire, *RR* relative risk, *QoL* quality of life, *OR* odds ratio, *MDADI* MD Anderson dysphagia inventory, SD standard deviation

## Discussion

To the best of our knowledge, this is the first systematic review of the literature based on PICO criteria aimed to evaluate the role of acupuncture in the management of radiation-induced toxicity in head and neck cancer. The significant burden in terms of long-lasting side effects [51] and quality of life impairment [52, 53] inflicted by standard CRT on patients with HNSCC call into question the potential need to integrate non-pharmacologic supportive care measures for symptom control. In view of the growing consideration of acupuncture within the therapeutic armamentarium [54–56] for common systemic therapy related toxicities, and in recognition of the limited data [57] available in respect to radiation therapy, we sought to analyze the literature in the most rigorous way, aiming to provide evidence-based recommendations for clinical practice in head and neck cancer. Following our prespecified search strategy, only a limited number of articles qualified for qualitative analysis. We had initially planned to pool the study-specific measures of association between acupuncture and the outcomes of interest (radiation-induced toxicities) into summary estimates using random-effects meta-analysis models. However, this was not possible because of the too limited number of available estimates for some endpoints; the large heterogeneity in terms of study settings and, in particular, in the way the outcomes were defined and measured; and the variability in reporting the measures of association with acupuncture. Therefore, the salient features and the main findings of the studies included in the systematic review were presented in tables and commented narratively in the text, whereas no formal statistical analysis could be conducted. Over 70% of patients (447/633) included in our work were randomly allocated to receive acupuncture throughout RT or CRT to evaluate its potential preventive effect on xerostomia. Overall, the most relevant common finding in both Meng’s [46] and Garcia’s [49] papers was that the delivery of TA was significantly associated with less severe patient-reported symptoms of salivary impairment (by definition, a score of 30 or more with the validated 8-item Xerostomia Questionnaire) compared with standard supportive measures up to 1 year from the end of treatment. In addition, in terms of preserving stimulated salivary flow rate, similar results were reported by Braga [45] and Meng [46]. As already anticipated, caution should be advised when addressing these 3 studies [45, 46, 49] together, keeping in mind their low comparability due to large differences in terms of timepoints of assessment, methodology, limited follow-up time and heterogeneity in patient and treatment characteristics, in particular RT technique. In this regard, extensive radiobiological models [58] underpinned the critical dependence of parotid gland damage from radiation dose distribution. In the pivotal phase 3 randomized Parsport trial [23], a mean difference of 24.5 (99% CI  − 4.3 to 53.2; 59.3 to 34.8, *p* < 0.0001) was observed 24 months after RT on the EORTC HN35 dry mouth subscale score in favour of patients treated with IMRT over 3DCRT. Selective sparing of stem cells in parotid sub-regions [59], pre-treatment functional characterization through quantitative imaging [60] and machine learning applications [61] are active areas of research for improving the technical capability of xerostomia prevention. For this purpose, no pharmacological interventions are currently supported in the clinic with sufficient evidence [62–64]. Albeit not further replicated in prospective studies, the results reported by Simcock et al. [47] lend support to the potential symptomatic efficacy of acupuncture on radiation-induced late salivary impairment, with significant improvement on the rate of patient-reported severe dry mouth (OR 2.0, *p* = 0.031), sticky saliva (OR 1.67, *p* = 0.048), need to sip to swallow food (OR 2.08, *p* = 0.011), and in waking up at night to drink (OR 1.71, *p* = 0.013), over standard oral care. From a mechanistic perspective, limited data are available [27] to justify the relieving effect of acupuncture on xerostomia. In a prospective trial, 20 healthy volunteers were randomized to receive TA or SA in a blinded fashion. By assessing the correlation between needle manipulation at L1–L2 acupoint (radial side of the non-dominant hand), stimulated salivary flow rate and functional magnetic resonance imaging, Deng et al. [65] demonstrated a higher salivation (mean difference of 0.34 g) obtained with TA over SA. In addition, a fair correlation between saliva production and neuroimaging activation of specific neuronal circuitry (parietal operculum, rolandic operculum, frontal operculum and insula, bilaterally) was also shown (0.63 coefficient at linear regression). The existence of a different pattern of neuroimaging activation induced by specific acupoint stimulation for the head and neck area was corroborated by further observations [66, 67] in non-oncologic patients. In analogy to other symptoms, it was hypothesized that the mitigating effect of acupuncture on xerostomia may result from its modulation of systemic inflammatory cascade and of parasympathetic and sympathetic balance at a glandular level [68, 69].

As demonstrated by our study results, other than xerostomia, very limited data [43, 48, 70–72] are currently available on the role of acupuncture in respect to other treatment-related side effects in HNSCC. In particular, there is little evidence [48] regarding the potential preventive effect on radiation-induced dysphagia. At diagnosis, most patients with locally advanced HNSCC already suffer from a variable degree of swallowing impairment [73]: its baseline assessment and management during therapy are extremely important factors with potential implications on the therapeutic success [74]. The adoption of preventive measures such as behavioral swallowing interventions may be helpful, albeit associated with a generally modest rate of patient adherence [75] and unclear impact on patients’ quality of life [76], being supported by low-level evidence [77]. Currently, the most reproducible way to try to mitigate the effects of treatment on the swallowing function resides in the implementation of swallowing-sparing IMRT. In last 15 years, a large body of evidence [6, 78, 79] was published on the correlation between radiation dose to pharyngeal constrictor muscles and the risk of developing acute and late swallowing impairment. No pharmacologic interventions aimed at preventing radiation-induced dysphagia can be recommended in the clinic. With the aim to demonstrate a protective effect of TA on patient-reported dysphagia in terms of M.D. Anderson Dysphagia Inventory (MDADI) [80] change from baseline to 1 year after treatment, Lu et al. [48] were not able to show any difference between TA and SA arms (improvement from baseline: + 7.9 points in TA arm, 95% CI 0.2–15.6, and + 13.9 points in SA arm, 95% CI 6.4–21.4; *p* = 0.17 for the comparison, 95% CI − 14.7 − 2.7). However, only 34/42 patients (81%) completed the study follow-up visits, with a potential imbalance in terms of RT total dose delivery between the two groups (70 Gy delivered in 90 and 76% of TA and SA groups, respectively). Throughout CRT, the onset of dysphagia should be considered as a critical component of a multifactorial painful syndrome [81, 82]: in view of the known analgesic effect of acupuncture in cancer [32] and preclinical data [83, 84], additional evidence is required to elucidate its preventive potential on irradiated swallowing structures. For the time being, state-of-the-art parotid and swallowing-sparing IMRT must be regarded as standard of care in everyday’s clinical practice.

Overall, the results of our study reinforce the notion that acupuncture is feasible in conjunction with anti-cancer treatment even if intensive as concurrent chemo-radiation can be, being associated with a very favorable toxicity profile. As reflected by our data, there’s increasing interest in applying acupuncture in head and neck cancer [38, 72, 85]. Although a detailed description is not within the scope of this work, our findings indirectly confirm the shortcomings bound with performing clinical research in the field of acupuncture [50, 86–88]. In spite of the methodological rigorousness based on a PICO-literature search, the large heterogeneity found in the retrieved data restrained us from providing any definitive recommendation on the role of acupuncture in the field of HNSCC. We acknowledge that focusing on papers published in languages other than English (i.e., Chinese) may represent a limitation of our study, as well as the exclusion of results obtained with ALTENS [89–91].

## Conclusions

In the context of locally advanced HNSCC, no evidence currently supports the routine use of acupuncture for the majority of acute and late radiation-induced side effects. Although shown to be feasible and safe in a single pilot randomized sham-controlled trial, no data currently justify its clinical application for the prevention of dysphagia. The large heterogeneity and low comparability of studies highlighting the impact of acupuncture on acute and late xerostomia don’t allow to provide any definitive recommendation in this regard. Further well-designed, controlled clinical trials should be warranted.

## Data Availability

Data supporting the findings of this study are available in the article or supplementary files.
